# Efficacy of three artemisinin combination therapies for the treatmentof uncomplicated *Plasmodium falciparum *malaria in the Republic of Congo

**DOI:** 10.1186/1475-2875-5-113

**Published:** 2006-11-24

**Authors:** Ingrid van den Broek, Christa Kitz, Sarwatt Al Attas, François Libama, Manica Balasegaram, Jean-Paul Guthmann

**Affiliations:** 1 Médecins sans Frontières, London, UK; 2 Médecins sans Frontières, Brazzaville, Republic of Congo; 3 Programme Nationale de Lutte contre le Paludisme, Brazzaville, Republic of Congo; 4 Epicentre, Paris, France

## Abstract

**Background:**

Presented here are the results of a comparative trial on the efficacy of three artemisinin-based combinations conducted from May to October 2004, in Pool Province, Republic of Congo.

**Methods:**

The main outcome was the proportion of cases of true treatment success at day 28. Recrudescences were distinguished from re-infections by PCR analysis. A total of 298 children of 6–59 months were randomized to receive either artesunate + SP (AS+SP), artesunate + amodiaquine (AS+AQ) or artemether + lumefantrine (AL), of which 15 (5%) were lost to follow-up.

**Results:**

After 28 days, there were 21/85 (25%) recurrent parasitaemias in the AS+SP group, 31/97 (32%) in the AS+AQ group and 13/100 (13%) in the AL group. The 28-day PCR-corrected cure rate was 90.1% [95% CI 80.7–95.9] for AS+SP, 98.5% [95% CI 92.0–100] for AS+AQ and 100% [95.8–100] for AL, thereby revealing a weaker response to AS+SP than to AL (p = 0.003) and to AS+AQ (p = 0.06). A potential bias was the fact that children treated with AL were slightly older and in better clinical condition, but logistic regression did not identify these as relevant factors. There was no significant difference between groups in fever and parasite clearance time, improvement of anaemia and gametocyte carriage at day 28. No serious adverse events were reported.

**Conclusion:**

Considering the higher efficacy of AL as compared to AS+SP and the relatively high proportion of cases with re-infections in the AS+AQ group, we conclude that AL is clinically more effective than AS+SP and AS+AQ in this area of the Republic of Congo. Implementation of the recently chosen new national first-line AS+AQ should be monitored closely.

## Background

In the Republic of Congo (RoC), malaria is the leading cause of morbidity and mortality in children under five years of age and the main reason for hospitalization. The total population of the country, totalling 3.2 million, is at risk of year-round highly endemic malaria. The percentage of medical consultations due to malaria is 30 to 50% [1,2]. According to the National Malaria Programme of the Ministry of Health, *Plasmodium falciparum *is responsible for 98% of malaria cases. The malaria incidence has increased over the past years, as a result of the deterioration of the health infrastructure and population movements during the country's recent episodes of conflict on the one hand and due to rising levels of resistance to common antimalarials on the other hand.

Chloroquine (CQ) was still the national recommended first-line and sulfadoxine-pyrimethamine (SP) the second line for uncomplicated falciparum malaria in 2004, at the time of study. Most recent data on drug efficacy have indicated a resistance as high as 90% against CQ and 25% against SP, and this was in a 14-day sentinel survey without PCR to identify new infections [3]. This seems to have rapidly increased since earlier data from 1999–2002 showed CQ resistance of 38–50% and SP resistance 0–10% [4,5]. Amodiaquine (AQ) resistance was also quite high in the few reported studies: 23% [6] and 21% in Brazzaville at day 7 [7].

The health authorities in the RoC were, at the time, preparing to change the national antimalarial treatment protocol, but still had to make a choice for the best suitable therapy. In most African countries, the first-line therapy has been revised recently and; at the time of writing, a total of 33 countries have chosen to implement artemisinin combination therapy or ACT, although only 14 have actually commenced implementation [8].

Here, we investigated the efficacy of three ACTs, that were considered potential new first-line therapies for the country, namely artesunate (AS) + SP, AS + AQ and artemether-lumefantrine (AL), in Pool Province in the southeast of RoC.

## Patients and methods

### Study location

**Figure 1:**
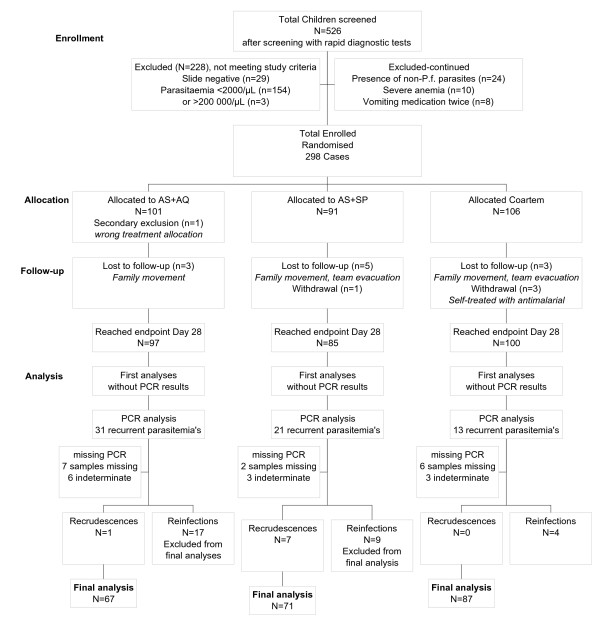
Flow chart drug efficacy trial.

Kindamba is the principal town of Kindamba district in the South of the RoC, located in Pool province (Figure 1). This is a heavily malaria-infested area, which was also the main battleground during the periods of internal conflict during the past decade. The security situation improved since the beginning of 2004 making it possible to execute a proper malaria drug efficacy trial (except for a three-week unsafe period when only a skeleton team remained for follow-up of study-patients, no new cases were enrolled). The population of Kindamba Health Zone was estimated at 20,000 but has increased with the improving security situation. Malaria tends to be holo-endemic the whole year through. The season for malaria is bimodal in the south, with a peak at the end of both rainy seasons from March to May and late September to December.

The health centre where the study was based is the *Kindamba Centre de Santé Intégré*, supported by MSF since 2003. This clinic saw about 500 to 600 cases each month of laboratory-confirmed malaria cases in 2004. The proportionate malaria morbidity among outpatients averaged to about 45% in the under 5's and 30% in older age-groups, while this was over 70% and 40% of in-patients for the two age groups, respectively.

### Study design

The standard WHO protocol for *in vivo *drug efficacy studies in areas of high transmission was followed [9]. Briefly, all children between 6 and 59 months of age presenting with fever (37.5°C or above) or a history of fever within the past 24 hours and a *P. falciparum *mono-infection with density between 2,000 and 200,000 parasites per μl were included in the trial. Written informed consent was asked from the care-taker of the child. Excluded were cases presenting with one or more general danger signs or signs of severe or complicated malaria [10], a serious concomitant illness, malnutrition or a known hypersensitivity to one of the study drugs, and those living further than 2 hours walking distance. In addition, children weighing less than 10 kg were not included in the group treated with AL, in accordance with then existing prescription guidelines in 2004. For the other two treatment groups weight limits were set at 5 kg, considered a minimum weight for 6 month-old infants.

Children were enrolled in the study and randomly assigned to one of three therapies: (1) AS + SP: single dose SP on the first day (Fansidar^®^, La Roche, France; 25 mg S and 1.25 mg P per kg) and 3-day AS (Arsumax^®^, Sanofi, France; 4 mg/kg body-weight per day), (2) AS + AQ 3-day AQ (Camoquin^®^, Parkes Davis/Pfizer, UK, 10 mg/kg per day) and AS (4 mg/kg per day) and (3) AL fixed combination (Coartem^®^, 20 mg arthemeter + 120 mg lumefantrine, Novartis Pharma, Switzerland). The dosage of AL (twice daily for three days) depended on body weight and followed the manufacturer instructions. All were treated orally under supervision in the clinic. However, in the AL group, for patients that were unable to stay or to come back to the clinic in the evening, the second daily dose was taken at home and observed by village fieldworkers who visited the patient at the time of treatment. At each dose, AL was given with some fatty food or a glass of milk. When a child was assigned to AL but weighed below 10 kg, the next possible treatment allocation was given and AL was given to the following study patient.

Patients were asked to come back for treatment the following 2 days and for follow-up visits on day 3, 7, 14, 21 and 28. They were clinically examined at all visits, both to check for signs of illness as for potential side-effects of treatment, as passively reported to the medical responsible, and had their blood-film taken on all these occasions except day 1. Blood films (thin and thick smears) were stained by Giemsa and examined by experienced microscopists. A quantitative parasite count of asexual forms was performed for positive cases (using the parasite/White Blood Cell Count) and the presence of gametocytes was noted. Based on clinical and laboratory findings, cases were classified as early treatment failures (ETF), late clinical failures (LCF), and late parasitological failures (LTF) or adequate clinical and parasitological responders (ACPR, see WHO 2003c). The final outcome parameter of the efficacy trial was the cure rate at the end of the follow-up period of 28 days in each of the three treatment arms. A true failure was defined as a recrudescence defined by PCR analysis eliminating re-infections. Haematological recovery was assessed by haemoglobin measurements (using the Hemocue^®^, Ängelholm, Sweden) before and after treatment. The rescue treatment in case of failure was quinine for 7 days.

The PCR genotyping used for distinguishing recrudescences and re-infections was performed at the laboratory of the Shoklo Malaria Research Unit, Thailand, based on a previously described protocol [11], comparing the *P. falciparum msp-1*, *msp-2 *and *glurp *gene loci of pre- and post-treatment sample pairs. The outcome of treatment with PCR correction was based on the number of true recrudescences, excluding cases of novel infections or indeterminate PCR from analysis (as per protocol [9]).

Laboratory quality control was carried out on 10% of the slides by qualified laboratory experts working in laboratories within the other MSF projects in the country (Kinkala, Nkayi, Moussaka). Slides were read blindly, and results were compared afterwards with those from the study laboratory. Data analysis was performed using Excel database and SPSS software. We used χ^2 ^tests to compare categorical data (Fisher exact for groups smaller than n = 5). Continuous data were tested for normality (Test for Skewness, Shapira – Wilkoxon Test for Normality). Normally distributed data were analysed with T-tests and ANOVA, and not-normally distributed data with Ranksum tests. Multivariate regression analysis was performed to determine whether baseline characteristics were associated with the outcome of treatment. Age, weight, temperature and parasite density were entered into the model as linear variable or classified in 3 to 5 categories and haemoglobin level in 3 classes: moderate (5–7.9 g/dl), mild (8–10.9 g/dl) or no anaemia.

### Ethical approval

The protocol received ethical clearance from the RoC Ministry of Health of the Republic of Congo and the external Ethical Review Board used by MSF. The necessary permission from the local authorities (Sous-préfet and hospital senior staff) in Kindamba, Pool District of study was obtained.

## Results

### Baseline characteristics

From 27 May to 6 October 2004, a total of 298 children were enrolled in the study and 101 children received AS+AQ, 91 received AS+SP and 106 received AL.

Due to the different weight-entry criteria for AL (≥10 kg) as compared to the other two ACT groups (≥5 kg), the children in the three treatment groups were not equal for all basic demographic characteristics on admission. There was a significant difference in weight, age, MUAC and blood haemoglobin value between the AL group and the other two groups. 36.5% of children were below 2 years; the average age was 27 months (Table 1).

**Table 1:** Baseline characteristics of study participants, Kindamba, ROC 2004

	**AS + AQ**	**AS + SP**	**Coartem**	**Total**	**P-value**
**Number of patients** **(% of total)**	101 (33.9)	91 (30.5)	106 (35.6)	298	
**Sex: F/M (% F)**	46/55 (45.5)	38/53 (41.8)	50/56 (47.2)	134/164 (45.0)	P = 0.74
Age* (months)	20.5 ± 10.7 (7–48)	23.0 ± 11.9 (6–54)	36.1 ± 11.1 (12–59)	26.8 ± 13.2 (12–59)	P < 0.00 1
Weight*(Kg)	9.7 ± 2.3 (6–16)	10.1 ± 2.4 (5–19)	12.7 ± 2.0 (10–20)	10.9 ± 2.6 (5–20)	P < 0.00 1
MUAC* (mm)	142.5 ± 13.8 (112–180)	143.5 ± 11.8 (100–188)	151.5 ± 10.6 (130–190)	146.1 ± 12.7 (100–190)	P < 0.00 1
Haemoglobin* (g/dl)	8.8 ± 1.7 (5.0–12.8)	9.1 ± 1.7 (5.1–12.3)	9.6 ± 1.7 (5.1–14.2)	9.2 ± 1.7 (5.0–14.2)	P = 0.00 3
**Moderate anaemia **‡ **(Hgb 5 < 8 g/dl)**	30 (29.7)	25 (27.5)	16 (15.2)	71 (23.9)	P = 0.03
**Mild anaemia **‡ **(Hgb 8 < 11 g/dl)**	59 (58.4)	53 (58.2)	66 (62.9)	178 (59.9)	P = 0.80
Temperature *(°C)	38.0 ± 1.2 (35.8–40.0)	37.8 ± 1.3 (34.8–40.7)	37.9 ± 1.3 (34.8–40.7)	37.9 ± 1.3 (34.8–40.7)	P = 0.32 0
Parasite density** (per μl)	28,941 (2040–200,000)	24,997 (1200–200,000)	23,997 (2000–200,000)	25,890 (1200–200,000)	P = 0.65 1
**Gametocytes **‡	23 (22.8)	24 (26.4)	9 (8.5)	56 (18.8)	P = 0.00 3

* Values given as mean ± SD (Standard Deviation) and range (min-max value),

** Parasite density given as geometric mean and range.

‡ Anaemia/gametocytemia described by number + proportion of cases with anaemia/gametocytes.

The results of the external quality control of 300 slides revealed no disagreements in presence/absence of *P. falciparum *parasites. There were 4 slides that had different findings for *P. falciparum *gametocytes and in one slide the reference laboratory reported the presence of *Plasmodium ovale *parasites. None of these disagreements, nor some discrepancies found in parasite densities, changed the outcome of the treatment.

**Figure 2:**
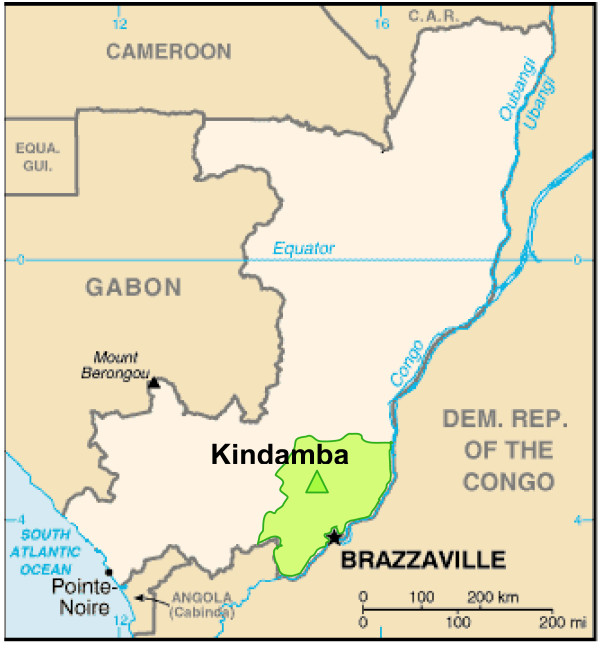
Map of the Republic of Congo showing the location of the study site, Kindamba, in Pool Province.

In total, 282 patients reached the study endpoint (94.6%), 11 were lost to follow-up (due to migration out of the area) and 5 were secondary exclusions (1 was retreated while not (yet) a failure, 4 were given drugs at home with antimalarial activity during follow-up; see Figure 2). There was no significant difference between the cases lost to follow-up or withdrawn in the groups AS + AQ (4%, 4/101), AS + SP (7%, 6/91) and AL (6%, 6/106, P = 0.42).

#### Primary treatment outcome

The proportion of recurrent parasitaemia's during the 4-week follow-up was higher in the AS+AQ group (32.0%; 31/97), and AS+SP group (24.7%; 21/85) than in the AL group (13.0%; 13/100). No ETF's were observed in any of the three treatment groups.

A total of 57 patients were excluded after PCR analysis. From the 65 patients eligible for PCR analysis, only 50 PCR were performed (15 samples not taken during a security evacuation of part of the study team). Among these, only 38 had a PCR result available (12 indeterminate results), of which 30 were re-infections which were excluded from the final analysis (Table 2; Figure 2). The proportion of re-infections was 18% (17/97) in the AS+AQ group, 11% (9/85) in the AS+SP group and 4% (4/100) in the AL group. The re-infection rate after AS+AQ treatment was significantly higher than after AL (p = 0.002).

**Table 2:** Proportion of recurrent parasitaemia and treatment failures at 28 days after treatment, Kindamba, ROC, 2004

	**AS + AQ**			**AS + SP**			**AL**			**χ^2^**
	**n**	**%**	**95% CI**	**n**	**%**	**95% CI**	**n**	**%**	**95% CI**	**P**

**No PCR**	**97**			**85**			**100**			
ACPR	66	68.0	57.8–77.1	64	75.3	64.7–84.0	87	87.0	78.8–92.9	0.006
Parasites/fever	19	19.6	12.2–28.9	9	10.6	5.0–19.1	5	5.0	1.6–11.3	
Parasites/no fever	12	12.4	6.6–20.6	12	14.1	7.5–23.4	8	8.0	3.5–15.2	

**Recurrent parasitaemia**	31	**32.0**	22.9–42.2	21	**24.7**	16.0–35.3	13	**13.0**	7.1–21.2	0.006

**PCR adjusted**	**67**			**71**			**87**			
ACPR	66	**98.5**	92.0–100	64	**90.1**	80.7–95.9	87	**100.0**	95.8–100	0.003
LCF	0	0.0	0–5.4	2	2.8	0.3–9.8	0	0.0	0–4.2	
LPF	1	1.5	0–8.0	5	7.0	2.3–15.7	0	0.0	0–4.2	

**Treatment failure**	1	**1.5**	0.0–8.0	7	**9.9**	19.3–4.1	0	**0.0**	0.0–5.2	0.003

At day 28, the AS+AQ group showed a cure rate of 98.5 [95% CI 92.0–100], the AS+SP group 90.1% [95% CI 80.7–95.9] and the AL group 100.0% [95% CI 95.8–100.0]. The cure rate of the AS+SP group was lower than that of the AL treatment (p = 0.003), and AS+AQ (p = 0.06) whereas differences in efficacy between AL and AS+AQ were not significant (p = 0.4).

Cases of recrudescence as well as cases of re-infection occurred at a relatively long time interval after treatment in all three groups. The median time to recurrent parasitaemia was 21 days for the AS+AQ group, 21 for AS+SP and 28 for AL.

Multivariate regression analysis showed that for 'recurrent parasitaemia', none of the baseline characteristics age, sex, weight initial parasite count, temperature or haemoglobin level influenced the outcome significantly; independent of these, the treatment group was the primary determinant of recurrent parasitaemia at day 28. For the recrudescent cases (PCR corrected true failure rates), multiple factorial analysis was not possible, due to the low numbers of failures.

#### Secondary treatment outcomes

On inclusion, 178/298 (60%) of patients had measured fever. The axillary temperature decreased rapidly after all three therapy regimens. At day 3, the number of febrile patients was similarly low in each arm (ASAQ = 3/101, ASSP = 4/90, AL = 8/105). All children were parasite free on day 3.

**Figure 3:**
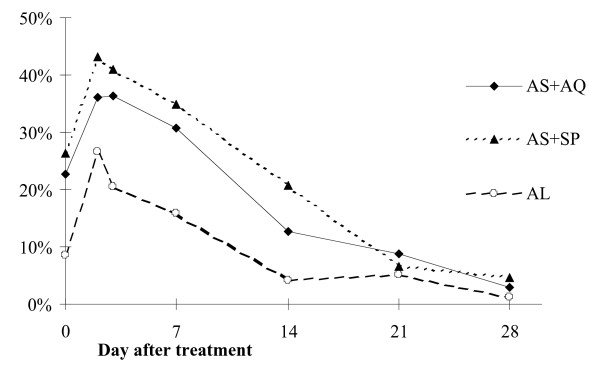
Gametocytaemia per treatment group during follow-up.

The proportion of cases with gametocytes increased during the first two days of treatment, but overall reduced during the four weeks of follow-up, from 23% to 3% in the AS+AQ group, from 26% to 5% in the AS+SP group and from 8% to 1% in the AL group (Figure 3).

**Figure 4:**
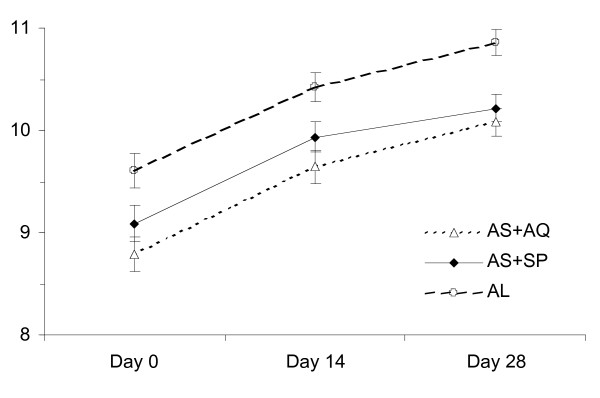
Haemoglobin values measured at 2-weekly visits in the three treatment groups.

There was a clear increase in the average haemoglobin levels in the three treatment groups during the 28 days of follow-up (Figure 4). Overall, the percentage of cases with moderate anaemia (5–7.9 g/dl) reduced from 23.9% at enrolment to 4.2%, while the proportion mildly anaemic (Hgb 8–10.9 g/dl) was still relatively high at the end of study, 61.9% compared to baseline 59.9%.

There were no clinically severe adverse events related to the treatment given. The most frequent complaints during the three days of treatment were vomiting, diarrhoea, abdominal pain and anorexia. The frequency of these potential treatment-related signs and symptoms was low (around 10% of children) and did not differ among the three treatment groups. There were two cases of urticaria, one after AS+SP and one after AS+AQ treatment, but these developed after completion of the treatment, so it was not necessary to stop or interrupt the treatment.

## Discussion

The study presented here shows that in the Pool region, Republic of Congo, AS+SP, AS+AQ and AL, are effective to treat uncomplicated *P. falciparum*. This is one of the first studies on ACTs in this country. AL is the most efficacious one of the three (100% with 95% CI 96–100); none of the children receiving this therapy had a recrudescent infection in the 4 weeks of follow-up. AS+SP showed a significantly lower efficacy than AL (90% with 95% CI 87–96), and AS+AQ (98.5 with 95% CI 92–100). The secondary outcomes of rapid fever-, parasite- and gametocyte clearance and the beneficial effect on blood haemoglobin levels were similar for the three therapies.

However, a substantially large proportion of children showed a recurrent parasitaemia in 4 weeks after treatment: 32% in the AS+AQ group, 25% in the AS+SP group and 13% in the AL group. AL appeared to prevent re-infection better than AS+AQ. Infection rates can be assumed equal among the treatment groups. AS is cleared from the blood within hours, so any protective effect of combination therapy comes from the remaining drug levels of AQ, SP and lumefantrine. Higher re-infection rates may be related to reduced AQ susceptibility/developing resistance of the parasites.

The interpretation of PCR analysis should be taken cautiously. Re-infections were confirmed in 46% of the recurrent infections, but there was a substantial part of PCR results unavailable (42% or 27/65), due to missing samples and indeterminate results. The available PCR results indicate that about 20% of recurrent infections were recrudescences; applying this 'rule' to the missing values, we calculated failure rates of 6% for AS+AQ (3 extra recrudescent cases assumed from 13 missing cases), 11% for AS+SP (1 added of 5 missing cases) and 2% for AL (2 added of 9 cases).

The treatment allocation was randomized, however, the specific entry criteria of weight for the AL group (above 10 kg) may have influenced the outcome of the study. The children selected for treatment with AL were on average older, and healthier. This group therefore may have had a better general condition and probably acquired some immunity against malaria, preventing rapid recrudescence, leading to a higher measured efficacy of AL. We had to follow the AL prescription criteria for ethical reasons, but maintained the usual weight limit for the other two treatment arms, to keep this study comparable to others in the region. The limitation of weight for AL has now been lowered from 10 kg to 5 kg in December 2004 by WHO [12,13].

The true failure rates may have been higher. Treatment failures can occur more than 28 days after treatment. It has been estimated [14] that 28-days assessments underestimate the true failure by about 20% for drugs with intermediate elimination half-life, such as lumefantrine and SP (3–6 days half-life [15]), and as much as 40% for drugs with a longer elimination half-life, such as desethylamodiaquine, the active metabolite that AQ quickly metabolises into (one to three weeks half-life [16]).

The levels of efficacy compare to findings from other recent studies on ACTs in surrounding in southwest Africa. In Angola, AL, AS+AQ and AS+SP were found to be highly effective [17]. In DRC, somewhat lower efficacy levels were found for AS+AQ, ranging from 81% to 93% while the findings for AS+SP were similar in one site, but lower, 67%-80% in other [18]. In Gabon, AS+AQ showed a 85% efficacy [19]. AL has shown a high efficacy where tested in Africa, in the range 94–100% [4,12,20].

Artemether-lumefantrine has shown more consistent efficacy to treat uncomplicated *falciparum *malaria than the other two ACTs in the study area in Congo Republic, and this is reflected by studies in the neighbouring countries as well. A careful implementation of AL is, therefore, recommended as the new protocol.

However, recent decisions at the National Malaria Programme in Brazzaville point towards AS + AQ as the new first-line antimalarial treatment, described for use at household as well as health centre level with basic symptomatic diagnosis (draft document PNLP). The second-line treatment defined is AL (or quinine or artemether) based on microscopy diagnosis.

Malaria is a high burden on health expenditures in Congo, a country with an infection prevalence estimated at 50% or more [21], leading to an incidence of about one episode per year in children < 15 years old [22]. The price of AL as compared to AS + AQ has probably played a significant role in the decision-making, as well uncertainties on the availability of AL at large scale. Congo is a low-income country and close to 50% of the people live under the limit of poverty (1 US$ per day), so the increase in cost of antimalarial treatment with the introduction of ACT cannot depend on patient fees. Treatment should be provided at subsidized cost from government health facilities, and external funding will be needed to realize that. In addition, the country has a largely urban population: 60% of the people live in town, the majority in Brazzaville and Pointe Noire. For this group 'formal public health facilities are often the last source of treatment used along the pathway to cure' [23] and this poses additional challenges to deliver a new ACT therapy and impose correct prescription.

## Conclusion

Implementation of the new RoC anti-malarial policy should be accompanied by adequate population sensitisation and facilitation of clear prescriptions using available blister-packaging or co-formulation, to prevent misuse of valuable medication risking further development of AQ resistance. The efficacy of the artemisinin combination therapies should be monitored at 2-year intervals after implementation.

## Authors' contributions

I Van den Broek was overall study supervisor and co-author for the final report. C Kitz was the site investigator in the field and co-author. S Attas contributed to the study implementation and was also overall medical coordinator for the MSF field programme. F Libama reviewed the study protocol and provided technical laboratory assistance and support. M Balasegaram and JP Guthmann were both involved in the study design, providing technical assistance and contributed to the writing of the final report.
